# Remarks on the Use of the Nitrate of Silver in Leucorrhea

**Published:** 1851-11

**Authors:** 


*Remarks on the Use of the Nitrate of Silver in Leucorrhea.* By Napoleon B. Anderson, M. D., of Louisville, Ky.

In the practice of medicine, the physician encounters many forms of disease which baffle all the resources of his art. Among those which have been a fruitful source of disappointment and mortification to the young practitioner, *Leucorrhea* is conspicuous. It has been treated by every conceivable mode, and in a majority of cases all the usual remedies have failed to relieve it. I have seen the lancet tried, with spare diet, laxatives, and the antiphlogistic generally. I have seen it treated with astringents and tonics, local and general — have seen different patients take calomel, guiacum, copaiba, cantharides, arnica, lime-water, myrrh, bark, rhubarb, steel, sarsaparilla, uva ursi, iodine, opiates, diuretic salts, cicuta, kino and the balm of gilead; seen them use injections of nitrate of silver, tannin, alum, sulphate of copper and zinc; seen the warm and cold hip-bath resorted to, and all without effect. The inference is irresistible — the complaint has been treated empirically. We have been groping in the dark, without any clear conception of the morbid condition giving rise to the symptoms. We have been treating a symptom instead of a pathological state. As long as we pursue this course we shall be disappointed in the result of our curative efforts. But there is no longer an excuse for adhering to a blind routine practice in this complaint; we possess the means for acquiring precise, accurate knowledge concerning its seat, cause, and character. The *speculum* enables us to do with the parts in which it has its origin what the unaided eye can do in the case of disease of external organs.

And yet, strange as it may appear, there are physicians who deprecate the use of this instrument. There are those who, with all the substantial aid it furnishes us, would proscribe its employment in every case. They maintain that it is indelicate; that it is shocking to female modesty, and hurtful to female virtue, and what we gain over the maladies of our fair countrywomen society loses in morality. They fall in with the popular prejudice, which would confine obstetrical practice to the hands of females. They would have none midwives but women.

Now all this must strike sensible men as eminently absurd. Nothing pursued with reference to the promotion of science can be immoral in its tendency. Female modesty is in no more danger from examinations looking to disease than from the manipulations necessary in parturition. If it has gone unharmed through the long centuries in which male midwifery has been practiced, it may be presumed that it will not be damaged by the use of the speculum.

Leucorrhea is always a secondary affection. In the healthy state of the parts, the vagina and cervix-uteri are lubricated with a fluid secreted by the lacunæ on their surface, and a serous exhalation from the membrane lining the cavity of the uterus; but while the parts are healthy, the absorption and secretion are so nearly balanced that no external discharge takes place. In *fluor albus*, there is perverted as well as augmented secretion. There is increased sensibility of the parts, and a bearing down sense about the uterus, with pains, in some cases, in the rectum, the bladder, the thighs, and the back. It is often attended with prolapsus uteri, and perhaps as often depends upon ulceration of the cervix. And how are we to make a correct diagnosis of each case? Shall we trust to the old methods, or call in the aid of the speculum? I deem that few practitioners will hesitate to avail themselves of all the light they can command in such circumstances.

In the course of the last year I have had an opportunity of treating forty-one cases of leucorrhea, and the results have been so satisfactory that I feel justified in submitting my experience to the profession. I am sure that no physician, after trying the improved method, will ever be satisfied to return to the old practice of tonics and astringent injections. Not that tonics are not useful in their proper place. I have found them highly beneficial in many cases, but they are to be looked upon as adjuvants to the main remedy — the nitrate of silver locally applied. So long as the morbid condition giving rise to the unhealthy secretion continues, constitutional remedies are of no avail. The cause must first be removed, and at a knowledge of this, as has already been remarked, we must arrive by means of the speculum.

In nine cases out of ten which have come under my treatment, I have found no difficulty in obtaining the consent of the patient to the use of the speculum: the woman is then placed in a horizontal position, on a lounge or bed, the hips elevated higher than the head; a loose sheet is thrown over her person, in which is an aperture sufficient to admit the speculum; (that which I use is glass;) it is well lubricated, and then insinuated as far as possible into the vagina, following the axis of the pelvis; — the os uteri readily descends into the mouth of the instrument, inclining backward and downwards, which, by depressing the handle, will remain in the centre of the instrument. In this position, before a window, or with the use of a candle, the organ can be viewed to its full extent, and the ulcerated surface found. In nearly every case it presents the same appearance. The neck is found flabby, swollen, and of a pale red and bluish cast — the anterior and posterior lips being much elongated, with the greater portion of the neck ulcerated to some extent. In about one-half of the cases, the ulceration is within, as well as without the neck, and extends some distance into the cavity of the uterus; whilst in the remaining half, it is limited to an area of one-third of the neck, and anterior and posterior lips, the cavity not partaking of the ulceration. In every case, the ulcerated surface is to some extent the same, with but little sensibility about the parts; no pain is ever felt on applying the caustic freely, both around the neck and within the uterine cavity, for the mouth is always open sufficiently to admit of its introduction.

In the first place, the parts are cleansed with a soft sponge attached to a whale-bone handle, in mop shape, after which the nitrate of silver, in the solid form, is applied very freely to every part diseased. This operation is repeated according to indications, from three to seven days apart, and during the interval, cold water injections several times a day are to be used conjointly with tonics. In some cases I have used the speculum as often as ten times, but in the great majority of cases, it is not necessary to resort to it often.

Those cases in which the ulceration was seated within the neck and cavity of the uterus occurred in weakly females, who had borne a great number of children, and some with the aid of forceps. In unmarried ladies, it was always seated on the external parts of the neck, and the anterior lip. In the cases in which the cavity was implicated, the free use of the silver was not found to have interfered in the least with the catamenia.

In almost every case where a female does not bear children, I have found fluor albus to be present; — unquestionably this disease is a frequent cause of sterility — the ulceration of the neck and cavity interfering with the intromission of the semen, which passes away with the profuse discharge.

September, 1851. — *Western Jour. of Med. and Surgery.*

